# Are auditory cues special? Evidence from cross-modal distractor-induced blindness

**DOI:** 10.3758/s13414-022-02540-0

**Published:** 2022-07-28

**Authors:** Lea Kern, Michael Niedeggen

**Affiliations:** Department of Education and Psychology, Division General Psychology and Neuropsychology, Freie Universität Berlin, Habelschwerdter Allee 45, 14195 Berlin, Germany

**Keywords:** Distractor-induced blindness, Attention, Cross-modal perception, Inhibition, Multisensory enhancement

## Abstract

A target that shares features with preceding distractor stimuli is less likely to be detected due to a distractor-driven activation of a negative attentional set. This transient impairment in perceiving the target (distractor-induced blindness/deafness) can be found within vision and audition. Recently, the phenomenon was observed in a cross-modal setting involving an auditory target and additional task-relevant visual information (cross-modal distractor-induced deafness). In the current study, consisting of three behavioral experiments, a visual target, indicated by an auditory cue, had to be detected despite the presence of visual distractors. Multiple distractors consistently led to reduced target detection if cue and target appeared in close temporal proximity, confirming cross-modal distractor-induced blindness. However, the effect on target detection was reduced compared to the effect of cross-modal distractor-induced deafness previously observed for reversed modalities. The physical features defining cue and target could not account for the diminished distractor effect in the current cross-modal task. Instead, this finding may be attributed to the auditory cue acting as an especially efficient release signal of the distractor-induced inhibition. Additionally, a multisensory enhancement of visual target detection by the concurrent auditory signal might have contributed to the reduced distractor effect.

**Supplementary Information:**

The online version contains supplementary material available at 10.3758/s13414-022-02540-0.

## Introduction

In our daily lives, we are constantly surrounded by a vast number of stimuli deriving from different sensory modalities. The perceptual system with its limited capacity has to filter the incoming information and efficiently select stimuli relevant to the current task, while irrelevant, distracting stimuli need to be inhibited (Feldmann-Wüstefeld & Vogel, 2019; Hasher et al., 2007; Moher et al., 2014). In addition to bottom-up stimulus properties, this selection and inhibition is assumed to be controlled top-down by *attentional sets* (Desimone & Duncan, 1995; Olivers & Meeter, 2008). While target features are stored in positive attentional sets, or target templates, which enhance processing of stimuli matching this template (Dombrowe et al., 2011; Leber & Egeth, 2006), negative attentional sets, or distractor templates, contain features that attention is directed away from (Arita et al., 2012; Olivers & Watson, 2006; Woodman & Luck, 2007; Zhang et al., 2009). The features of target and distractors are not always distinct but can overlap, leading to impaired target processing if a negative attentional set containing these shared features is activated (Boncompte & Cosmelli, 2018; Folk et al., 2008; Lleras et al., 2008; Sahraie et al., 2001; Wu & Fu, 2017).

One experimental paradigm demonstrating the consequences of a negative attentional set comprising shared features of distractors and target-on-target detection is *distractor-induced blindness* (DIB). In the DIB task, participants detect a target (e.g., episode of coherent motion in random dot kinematogram) that is indicated by a cue (e.g., color change of fixation to red) (Sahraie et al., 2001). In the visual modality, it was observed consistently that target-like but task-irrelevant events (i.e., distractors) occurring before the cue are associated with a transient deficit in detecting the target. This “blindness” is most pronounced if cue and target are displayed simultaneously and vanishes at a cue-target stimulus-onset asynchrony (SOA) of 200–300 ms (Hesselmann et al., 2006; Sahraie et al., 2001; Winther & Niedeggen, 2018). The DIB effect has been attributed to a central inhibition of distractor (and therefore target) features, building up cumulatively with the repeated presentation of to-be-ignored distractors (Niedeggen et al., 2012; Niedeggen et al., 2015; Winther & Niedeggen, 2017b). Within this model, the cue is working as a release signal of the inhibition, leading to a gradual deactivation of the negative attentional set and recovering target detection rates with longer cue-target SOAs (Michael et al., 2011). A higher selection difficulty of the cue due to lower salience is associated with a larger DIB effect (Hay et al., 2006). Previous research ruled out that DIB can be accounted for by spatial shifts of attention between the two visual streams containing cue and target (Hesselmann et al., 2009).

To date, DIB has been observed for the features motion, orientation, and color (Michael et al., 2011; Winther & Niedeggen, 2017a). Some models of attentional control assume that inhibition of irrelevant stimuli works in a similar fashion for different visual features (Hasher et al., 2007; Olivers & Meeter, 2008). This assumption is supported by neuroimaging studies suggesting that working memory processes including inhibition do not seem to be organized by stimulus type (for a meta-analysis, see Wager & Smith, 2003). Interestingly, one difference between visual features was demonstrated for DIB: color changes are apparently more effective in eliciting the inhibitory process than motion stimuli (Winther & Niedeggen, 2017a; Winther & Niedeggen, 2018). This finding might be attributed to differences between ventral and dorsal stream processing (Winther & Niedeggen, 2017a), with color being predominantly processed in the ventral and motion/orientation being primarily associated with the dorsal visual system (e.g., Kravitz et al., 2013; Valyear et al., 2006). The distinction between ventral and dorsal processing has been proposed to not only apply to perceptual processing stages but also to working memory selection (Nee et al., 2013).

The effect of distractors on target detection is not restricted to the visual modality. Recent studies revealed a *distractor-induced deafness* (DID) (Kern & Niedeggen, 2021a), which can also be observed under cross-modal stimulation (Kern & Niedeggen, 2021b). In the previous cross-modal set-up (Kern & Niedeggen, 2021b), the cue was presented in a rapid serial visual presentation (RSVP) sequence and the target occurred in an auditory stream. The visual sequence consisted of a circular presentation of eight grayscale bars, giving the impression of a circular movement (“preloader” symbol). A small white circle with a black outline could appear at the position of one of the bars, defining the cue. The target was a short rise in amplitude in a continuous tone occurring with or after the cue, while rises in amplitude before the cue were distractors that ought to be ignored. In accordance with unimodal findings, multiple distractors impaired target detection, especially if cue and target appeared concurrently. Interestingly, DID in both the uni- and cross-modal settings was associated with a smaller decrease in target detection compared to the visual modality. However, the DIB effect remained to be examined for the combination of auditory cue and visual target.

The processing and detection of target stimuli in a *multisensory*, or *cross-modal*, environment is in addition to attentional selection and inhibition processes observed within modalities also influenced by interactions between the senses (Koelewijn et al., 2010; Spence et al., 2009; Stein & Stanford, 2008). If two stimuli from different modalities overlap in presentation time and/or spatial location, performance enhancements (i.e., *multisensory enhancement*) can often be observed compared to unimodal settings (Klapetek et al., 2012; Stein & Stanford, 2008; Stevenson et al., 2014; Talsma et al., 2010; Tang et al., 2016). Previous research demonstrated that an auditory stimulus temporally coinciding with a visual target can cause an enhancement of perceived visual stimulus intensity (Noesselt et al., 2008; Stein et al., 1996) and of target detection rates (Andersen & Mamassian, 2008; Frassinetti et al., 2002; Giard & Peronnet, 1999; Gleiss & Kayser, 2013; Lippert et al., 2007; Noesselt et al., 2010; Van der Burg et al., 2011). Furthermore, multisensory (i.e., audiovisual) cues continue to capture attention under high perceptual load (Santangelo & Spence, 2007b; Spence & Santangelo, 2009), while effects of unimodal cues can be suppressed if task demands are high (Santangelo et al., 2007; Santangelo & Spence, 2007a). Consequently, potential influences of multisensory processing need to be considered in cross-modal settings.

The current study comprises three consecutive behavioral experiments that aimed to investigate whether the characteristics of distractor-induced blindness can be observed in a cross-modal setting. Here, the cue as signal of task relevance was defined in the auditory modality, while target and distractors were visual stimuli. More specifically, the following two characteristics should be examined: (1) are multiple target-like distractors associated with impaired target detection compared to zero and one distractor conditions? and (2) is the target detection impairment after multiple distractors most pronounced at a cue-target SOA of 0 ms, and decreasing with increasing SOA? If a cross-modal DIB effect with these characteristics can be stated, this in combination with previous findings (Kern & Niedeggen, 2021a, 2021b) speaks for DIB/DID occurring independent of the sensory modality cue and target are presented in. However, we also aimed to observe whether the modality cue and target appear in might have an impact on the expression of the distractor effect. Uni- and cross-modal DID showed a similar magnitude and appeared to be smaller than the visual DIB (Kern & Niedeggen, 2021a, 2021b). One possible reason for this observation could be that auditory distractors generally have a weaker influence on target processing, possibly due to differences in distractor processing between visual and auditory domain. If we find a cross-modal DIB of comparable size to the unimodal visual effect – therefore being larger than DID – this speaks for a larger impact of distractors on target detection in the visual compared to the auditory modality.

## Experiment 1

The aim of Experiment 1 was to examine whether two basic characteristics of distractor-induced blindness – the effect of multiple distractor episodes and the recovery of this effect as a function of the cue-target-SOA – can be found in a cross-modal setting with the cue embedded in an auditory and distractor/targets occurring in a visual stream. So far, such a distractor effect (distractor-induced blindness/deafness) has been shown within the visual (Sahraie et al., 2001) and auditory modality (Kern & Niedeggen, 2021a) and was recently also observed in a cross-modal task for a visual cue and an auditory target (Kern & Niedeggen, 2021b). In all of these settings, the largest distractor effect occurred at a cue-target SOA of 0 ms and decreased with increasing SOA. It remained to be examined if this effect also occurs in a cross-modal set-up containing a visual target.

The current study aimed to close this research gap by investigating cross-modal distractor-induced blindness with the cue as signal of task relevance being defined in the auditory modality (i.e., short rise in amplitude in a continuous tone) and target and distractors being visual stimuli (i.e., brief appearance of a small white circle). We expected to observe a decrease in target detection following the presentation of multiple distractors. The target detection deficit should be most pronounced if cue and target occur concurrently and should recover with increasing cue-target SOA. According to the DIB model, the transient “blindness” for the target is caused by the activation of a central inhibitory process by the distractors (Hesselmann et al., 2006; Niedeggen et al., 2015). Thus, one would assume that target detection should also be impaired in this cross-modal setting, irrespective of the cue being presented in another modality. To estimate the expression of the distractor-induced effect on target detection, the acquired data of Experiment 1 were compared to a previous behavioral experiment with visual cue and auditory distractors and target (Kern & Niedeggen, 2021b). If auditory distractors have a weaker influence on target detection than visual distractors, cross-modal DIB should show a larger magnitude than cross-modal DID.

### Method

The data, code, and stimulus material of all three experiments, which were not pre-registered, are provided in an open repository (10.17632/wxmhwv7xvd.1).

#### Participants

Sample sizes were calculated *a priori* using G*Power (Faul et al., 2007) for α = .05 and an intended power of 80% (F-test with repeated measurement). Based on a previous cross-modal study (Kern & Niedeggen, 2021b), we expected a large effect (*f* = .40) for the within-subject factor “number of distractors” (one vs. multiple distractors). This resulted in a required sample size of N = 15 for each of the three experiments.

Throughout Experiments 1–3, participants had no history of neurologic or psychiatric conditions, had normal or corrected-to-normal visual acuity, and self-reported normal hearing ability. Participants were recruited in the university environment, received course credit or 10€/h for participation, and gave written informed consent prior to testing. The experimental procedure (including Experiments 1–3) was approved in advance by the ethics committee of Freie Universität Berlin (027/2019). A priori defined exclusion criteria based on previous studies (Kern & Niedeggen, 2021b) were as follows: (1) unreliable task performance during pretest trials (< 60% correct target detection); (2) unreliable task performance during the experiment (< 25% hit rate in zero distractor condition at SOA 0 ms).

Eighteen healthy subjects participated in Experiment 1 (13 women; 20–35 years of age; *M*_*age*_ = 25.61 years, *SD* = 5.25). No participant had to be excluded based on the exclusion criteria. The data of Experiment 1 were compared to a previous behavioral data set (N = 20; 13 women; 18–39 years of age; *M*_*age*_ = 29.20 years, *SD* = 5.92) that examined cross-modal distractor-induced deafness and was published as part of Kern and Niedeggen (2021b).

#### Stimuli, procedure, and design

All visual and auditory stimulus material used in Experiment 1 was taken from previous experiments (Kern & Niedeggen, 2021a, 2021b). The resulting analogy in the set-up allowed a statistical comparison of the current data to a previous data set.

The experiment was conducted in a sound-attenuated chamber with dimmed and indirect lightning. Participants sat at 62 cm viewing distance in front of a 20-in. monitor (Sony Trinitron Multiscan G520) and wore in-ear headphones (Audio-Technica ATH-LS70iS) with individually fitted earpieces. Auditory stimuli were created and edited with the programs “Tone Generator” and “WavePad Editor” (NCH Software, Greenwood, USA). The experiment was run on a Windows PC using PsychoPy (Version 3.6.8 for Windows).

Participants heard two auditory sequences and monitored a dynamic visual stream concurrently. Trial duration was set to 5,000 ms. The two rapid serial auditory presentations (RSAPs) were each presented to one ear. On the right ear, a continuous tone with a modulation at 5 Hz in a frequency range from 270 to 330 Hz was played (stream 1). The auditory cue was defined in this sequence as a transient rise in amplitude (+10 dB) for 100 ms. The cue was presented at a randomized temporal position between 3,100 and 4,000 ms after trial onset. On the left ear, participants heard a second sequence consisting of 50 sine-wave tones (duration: 30 ms, inter-stimulus interval (ISI): 70 ms) that were each randomly selected from a set of seven tones (frequency range 1,800–2,200 Hz; stream 2). The amplitude of the continuous tone was reduced compared to the sine-wave tone sequence (-20 dB). Presentation time of the sine-wave tones was 30 ms with a 70-ms ISI between tones. In this second RSAP stream, no task-relevant event occurred. For the aim of comparability between current and previous cross-modal findings, we kept both auditory streams in the experimental set-up since removal of one of the RSAP sequences could affect task difficulty.

**Fig. 1:**
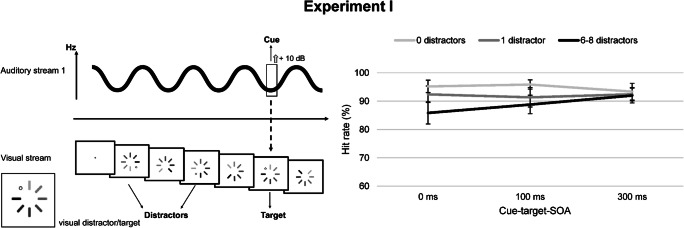
Task design and results of Experiment 1. *Note.* The left panel illustrates the task design: The cue was defined in the auditory modality, while target and distractors were presented embedded in a visual rapid serial visual presentation (RSVP) sequence. Target and distractor stimuli solely differed in the timing of their presentation: distractors appeared before cue onset, while a target could occur at the same time as or after the cue. The “preloader” symbol at the center of the screen changed every 100 ms, creating the impression of a clockwise motion. On the left ear, participants always heard a sequence of pure tones, while on the right ear a continuous tone was presented. In Experiment 1, the cue was a short rise in amplitude (+10 dB) in the continuous tone. The target event was the appearance of a small white circle within the preloader symbol for 100 ms simultaneous to or after the cue. The right panel shows the results of Experiment 1: Mean hit rates (in %; y-axis) are depicted for the three stimulus-onset asynchrony (SOA) conditions (x-axis) and the number of distractors. Error bars depict standard errors of the mean. Multiple distractors were associated with lower target detection rates at short cue-target SOAs

Simultaneous to the two auditory sequences, a RSVP was displayed at the center of the screen. Eight bars were arranged in a circle with a total retinal size of 1.66° in diameter. The impression of a clockwise motion was created by varying the luminance of adjacent bars, in analogy to the signal indicating a loading process in software or websites (“preloader” symbol). The lightest bar was colored in white and thus appeared to be missing, while the darkest bar was colored in black. Changes in luminance appeared each 100 ms with no ISI. Target and distractors were defined within this RSVP sequence: a small white circle with black outline (0.186° in diameter) could appear for 100 ms at the position of the missing bar in the preloader symbol. The occurrence of a small white circle at the same time as or following the auditory cue was defined as the target. Small circles appearing before cue onset were labeled as distractors and were to be ignored. Stimulus-onset asynchrony (SOA) between onset of the last distractor and cue was randomized between 500 and 700 ms. SOAs between distractors were set between 200 and 700 ms. If only one distractor was presented, it occurred at the temporal position of the last distractor in the multiple distractor condition (i.e., 500–700 ms before cue onset). The experimental set-up is depicted in Fig. 1.

After giving informed consent, testing started with 32 pretest trials, during which participants were familiarized with stimuli and task. The experimenter provided verbal feedback on the correctness of responses after each trial. If participants showed a good understanding of instructions and a reliable target detection during at least 60% of trials, the main experiment started. If task performance remained unreliable during pretest, testing was aborted (see exclusion criterion 1).

The experiment consisted of 290 trials, which were presented in randomized order for each participant. After each trial, participants had to decide whether they perceived an auditory cue (question 1: “Did you hear a change in the continuous tone?”) and whether they saw a target accompanying or following the cue (question 2: “Did you see a small white circle simultaneous to or after a change in the continuous tone?”). Non-speeded responses (yes/no) were given via button press on the keyboard. Participants were instructed to answer as accurately as possible. The experiment lasted approximately 1 h.

Targets could appear at the same time as or shortly after the cue (within-subject factor cue-target-SOA: 0 ms vs. 100 ms vs. 300 ms). Additionally, the number of visual distractors preceding cue and target was manipulated as second within-subject factor (0 vs. 1 vs. 6–8 distractors). For each SOA condition, 80 trials containing cue and target were presented. Within each of the three SOA conditions, 40 trials included multiple distractors, 20 trials had one distractor, and 20 trials comprised no distractors. To be able to assess the reliability of response behavior in terms of false alarms (falsely reported target after correctly detected cue), trials only containing the cue were added as a control condition (15 trials with multiple distractors, 15 trials without distractor). As a second control condition, 20 trials included neither cue nor target (ten trials with multiple distractor, ten trials without distractors).

The previous behavioral study, which examined cross-modal distractor-induced deafness and was compared to the results of Experiment 1, had used the identical auditory and visual streams, task-relevant stimuli, and experimental manipulations. Therefore, sensory stimulation was identical, and solely the assignment of cue and target to the sensory modalities was reversed (i.e., cue: appearance of small white circle; distractors/target: short rise in amplitude in continuous tone). The data of this previous experiment are accessible in an open repository (10.17632/6n7585w2jj.1).

#### Data analysis

The hit rate (i.e., correct target detection after correctly detected cue) was computed for each experimental condition and each participant. Behavioral data were analyzed using IBM SPSS Statistics 27 in a two-way repeated-measures ANOVA including the within-subject factors “SOA” between cue and target (0 ms vs. 100 ms vs. 300 ms) and “number of distractors” (0 vs. 1 vs. 6–8). Bonferroni-corrected post hoc comparisons were computed in case of a significant main effect or interaction. Greenhouse-Geisser corrections were applied if appropriate. If the interaction “SOA” x “number of distractors” reached significance, post hoc tests were conducted for each SOA to examine an influence of the number of distractors, and the hit rates in the multiple distractor condition were compared between the three SOAs to assess the recovery of hit rates.

In a second, additional step of analysis, the data acquired in Experiment 1 were compared to previous cross-modal behavioral data for reversed modalities (Kern & Niedeggen, 2021b) to assess a possible impact of the cross-modal setting. To this aim, an ANOVA with the repeated-measures factor “number of distractors” (0 vs. 1 vs. 6–8) and the between-subject factor “cross-modal setting” (auditory cue, visual target (Exp. 1) vs. visual cue, auditory target) was conducted, focusing solely on the cue-target SOA of 0 ms.

### Results

Mean target detection rates for Experiments 1–3 are presented in Table 1. The results of Experiment 1 are depicted in Fig. 1. Participants showed a reliable response behavior, as demonstrated by overall high hit rates and few false alarms (multiple distractors: *M* = 3.48 %, *SD* = 9.27; without distractors: *M* = 2.47 %, *SD* = 8.13). Occurrence of false alarms was not influenced by the presence of distractors, *F*(1, 17) = .95, *p* = .343, η_p_^2^ = .053. Furthermore, cue detection was reliable (*M* = 95.69 %, *SD* = 5.80 for multiple distractors, SOA 0 ms), indicating a high salience of the cue.

**Table 1:** Mean target hit rates and 95% confidence intervals (CIs) in Experiments 1–3

	Experiment 1			Experiment 2			Experiment 3		
Distractors	SOA 0 ms	SOA 100 ms	SOA 300 ms	SOA 0 ms	SOA 100 ms	SOA 300 ms	SOA 0 ms	SOA 100 ms	SOA 300 ms
0	*M* = 95.22	*M* = 95.87	*M* = 93.38	*M* = 94.42	*M* = 96.22	*M* = 95.05	*M* = 97.60	*M* = 97.41	*M* = 95.24
	CI [90.60, 99.83]	CI [92.42, 99.31]	CI [87.14, 99.63]	CI [88.56, 100.27]	CI [91.76, 100.67]	CI [91.53, 98.57]	CI [95.23, 99.97]	CI [93.69, 101.13]	CI [91.32, 99.17]
1	*M* = 92.42	*M* = 91.36	*M* = 92.44	*M* = 91.24	*M* = 92.51	*M* = 94.87	*M* = 95.24	*M* = 96.22	*M* = 97.11
	CI [86.23, 98.61]	CI [84.16, 98.55]	CI [87.56, 97.31]	CI [84.56, 97.91]	CI [86.89, 98.12]	CI [90.72, 99.01]	CI [91.78, 98.71]	CI [93.68, 98.75]	CI [94.43, 99.78]
6–8	*M* = 85.85	*M* = 88.82	*M* = 92.00	*M* = 85.79	*M* = 90.88	*M* = 90.10	*M* = 83.87	*M* = 90.76	*M* = 96.28
	CI [77.65, 94.05]	CI [81.94, 95.69]	CI [86.48, 97.52]	CI [78.90, 92.69]	CI [85.29, 96.46]	CI [83.34, 96.87]	CI [75.68, 92.05]	CI [85.19, 96.33]	CI [93.29, 99.26]

The main effect of number of distractors reached significance, *F*(1.15, 19.58) = 10.56, *p* = .003, η_p_^2^ = .283. Post hoc comparisons showed that hit rates decreased with increasing number of distractor events (0 vs. multiple distractors: *F*(1, 17) = 11.48, *p* = .003, η_p_^2^ = .403; 1 vs. multiple distractors: *F*(1, 17) = 14.53, *p* = .001, η_p_^2^ = .461; 0 vs. 1 distractor: *F*(1, 17) = 6.08, *p* = .025, η_p_^2^ = .263).

The SOA between cue and target had no significant effect, *F*(2, 34) = 1.28, *p* = .291, η_p_^2^ = .070. Instead, the interaction between “number of distractors” and “SOA” reached significance, *F*(4, 68) = 4.03, *p* = .005, η_p_^2^ = .192, indicating that distractors influenced hit rates differently at the three SOAs. Post hoc tests revealed a significant effect of number of distractors on target detection at short cue-target intervals (SOA 0 ms: *F*(1.24, 21.01) = 13.72, *p* = .001, η_p_^2^ = .447; SOA 100 ms: *F*(2, 34) = 6.27, *p* = .005, η_p_^2^ = .270), while this influence was extinguished at SOA 300 ms (*F*(2, 34) = .40, *p* = .673, η_p_^2^ = .023). Additionally, post hoc tests showed a significant recovery of target detection rates with increasing SOA for the multiple distractor condition, *F*(2, 34) = 7.38, *p* = .002, η_p_^2^ = .303, following a linear trend, *F*(1, 17) = 12.09, *p* = .003, η_p_^2^ = .416.

### Comparison between two cross-modal data sets: Does the cross-modal setting influence the distractor effect?

**Fig. 2:**
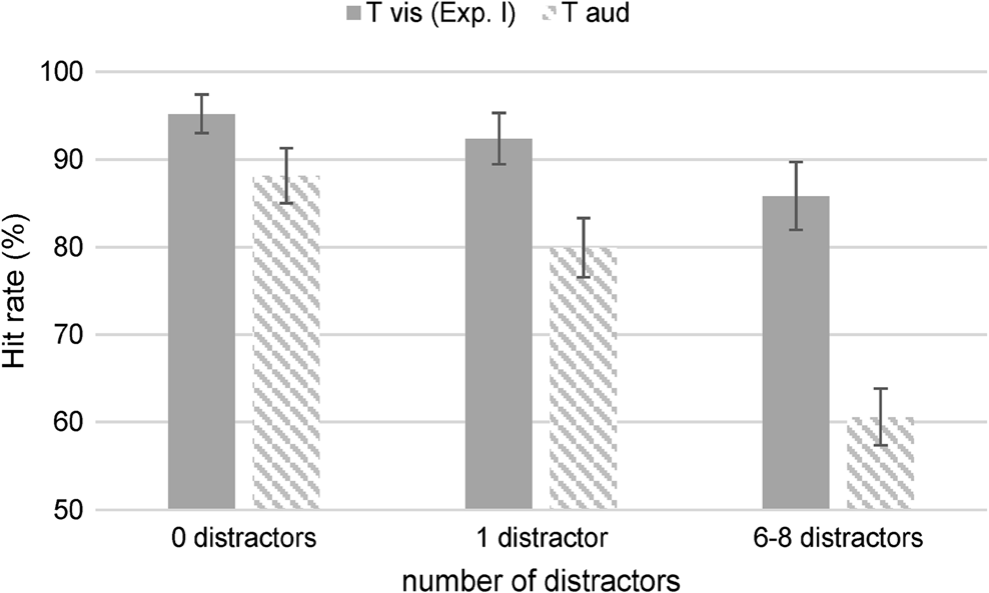
Distractor effects at cue-target stimulus-onset asynchrony (SOA) 0 ms in two cross-modal settings. *Note.* Hit rates acquired in Experiment 1 in comparison to hit rates from a previous cross-modal experiment (Kern & Niedeggen, 2021b) at cue-target SOA 0 ms. Both experiments had an identical set-up and sensory stimulation, and solely differed in the assignment of cue and target/distractors to sensory modalities. While target and distractors were visual and the cue auditory in Experiment 1, auditory distractors and target and a visual cue were presented in the previous study. Overall, hit rates were higher if the cue was auditory and the target visual (Experiment 1). A significant interaction between number of distractors and cross-modal setting could be observed, indicating a more pronounced drop in hit rates after the presentation of distractors if the target was auditory

In a following analysis step, the data from Experiment 1 and a previous cross-modal experiment with identical experimental set-up but reversed modalities regarding cue and target were jointly analyzed to examine an effect of cross-modal setting. Hit rates in both experiments at SOA 0 ms are displayed in Fig. 2. The analysis revealed a significant main effect of cross-modal setting, *F*(1, 36) = 9.35, *p* = .004, η_p_^2^ = .206, driven by higher hit rates in Experiment 1 (*M* = 91.16 %, 95% CI [83.97, 98.35]) than in the previous experiment with visual cue and auditory target (*M* = 76.22 %, 95% CI [69.41, 83.04]). Additionally, the interaction between “number of distractors” and “cross-modal setting” at cue-target SOA 0 ms yielded significance, *F*(2, 72) = 6.54, *p* = .002, η_p_^2^ = .154 (main effect number of distractors: *F*(2, 72) = 26.89, *p* < .001, η_p_^2^ = .428). A post hoc power analysis revealed that the estimated achieved statistical power for the interaction between the between-subject factor “cross-modal setting” and the within-subject factor “number of distractors” was 91.8%. Post hoc tests for each distractor condition showed no significant difference in hit rates if no distractors were presented, *F*(1, 36) = 3.24, *p* = .080, η_p_^2^ = .083, while higher hit rates were observed in Experiment 1 if distractors were present (one distractor: *F*(1, 36) = 4.53, *p* = .040, η_p_^2^ = .112, multiple distractors: *F*(1, 36) = 13.16, *p* < .001, η_p_^2^ = .268).

### Discussion

Experiment 1 demonstrated that a visual target presented concurrently with or shortly after an auditory cue is less reliably detected if it is preceded by multiple target-like distractors. As hypothesized, target detection was most impaired at a cue-target SOA of 0 ms. In line with the time course observed within the visual modality (Michael et al., 2011; Sahraie et al., 2001), the effect vanished at SOA 300, indicating the deactivation of the distractor template. Therefore, cross-modal distractor-induced blindness with the typical behavioral characteristics can be stated, providing evidence that a distractor effect cannot only be found within visual and auditory modality but also in both possible combinations between these modalities. The finding implies that target detection is reduced at SOA 0 ms in a cross-modal setting if a negative attentional set is activated by the repeated presence of multiple distractors in the auditory and visual domain. The release of this inhibition of visual features shows comparable properties for a visual and auditory cue.

Additionally, and in extension of previous research, the results of Experiment 1 demonstrate that DIB can also be elicited for the appearance of a small shape, which was associated with a local change in luminance. Consequently, a fourth visual feature could be established, which further strengthens the replicability of the DIB effect across different feature dimensions and speaks for a similar functioning of the inhibitory process for a variety of visual features.

While a substantial detection deficit for the visual target after multiple distractors and at short SOAs could be found, the size of the distractor effect (calculated as hit rate 0 distractor condition – hit rate multiple distractor condition at SOA 0 ms) was smaller than usually observed within the visual modality (Niedeggen et al., 2012; Winther & Niedeggen, 2017a). Furthermore, the comparison with a cross-modal data set from a previous study with a reversed assignment of modalities revealed that hit rates were higher if the cue was auditory and distractors and target occurred in the visual modality than vice versa. Importantly, the effect of distractors on target detection was more pronounced for cross-modal DID than for cross-modal DIB (see Fig. 2), indicating a greater susceptibility to distractors in case the cue was visual and the target and distractors auditory. This finding contradicts the assumption of a generally larger effect of visual compared to auditory distractors on the detection of a target in the same modality. That the cross-modal distractor effect appears to be reduced compared to the unimodal visual effect might be attributed to an enhancement of visual target processing by a concurrent auditory cue, which has been demonstrated for other paradigms (Frassinetti et al., 2002; Noesselt et al., 2008; Noesselt et al., 2010; Van der Burg et al., 2011; Zhao et al., 2020). This multisensory enhancement might counteract the distractor-induced inhibition to a certain degree. However, an alternative explanation could be that the cue feature rise in amplitude – and therefore a brief increase in loudness of the task-relevant auditory stream – might have been especially efficient in releasing the distractor-evoked inhibition and in redirecting attention to the visual target.

## Experiment 2

Sounds with increasing intensity in terms of loudness (i.e., “looming sounds”) can enhance neural excitability of low-level visual areas (Romei et al., 2009), as well as visual orientation sensitivity (Cecere et al., 2014; Leo et al., 2011) compared to sounds of constant intensity. Additionally, looming auditory stimuli have been associated with preferential processing during multisensory integration (Cappe et al., 2012; Sutherland et al., 2014) and are claimed to be very salient since they increase phasic alertness (Bach et al., 2009) and activate a distributed brain network associated with attentional processes (Seifritz et al., 2002). If a comparable process is triggered during cross-modal DIB, one could assume that the auditory cue characteristics affect the detectability of the target. Thus, the increase in loudness as cue might have triggered a fast attentional allocation to the target stream and could have initiated a more efficient release of the negative attentional set than a tone of constant intensity.

To test if the cross-modal DIB effect observed in Experiment 1 is influenced by the feature defining the auditory cue, Experiment 2 was conducted. This subsequent experiment included the identical visual RSVP stream containing target and distractors and the same auditory stimulation, except for a different auditory feature – frequency composition – now defining the cue. We expected to replicate the cross-modal DIB effect from Experiment 1. If a rise in amplitude as cue is an especially efficient release signal, the observed distractor effect at SOA 0 ms is expected to be larger in Experiment 2 compared to Experiment 1. For visual stimuli, previous studies that experimentally manipulated cue salience indicated that a more salient cue is associated with a smaller DIB (Hay et al., 2006). To test for potential differences in cue salience, cue detection rates were assessed and compared between both experiments.

### Method

#### Participants

Twenty-one new participants who did not participate in Experiment 1 were tested in Experiment 2. The same requirements, exclusion criteria, and a priori power analysis outlined for Experiment 1 applied for Experiment 2. One participant had to be discarded due to fulfilling the second exclusion criterion. The final sample consisted of 20 participants (12 women; 18–35 years of age; *M*_*age*_ = 25.59 years, *SD* = 4.46).

#### Stimuli, procedure, and design

**Fig. 3:**
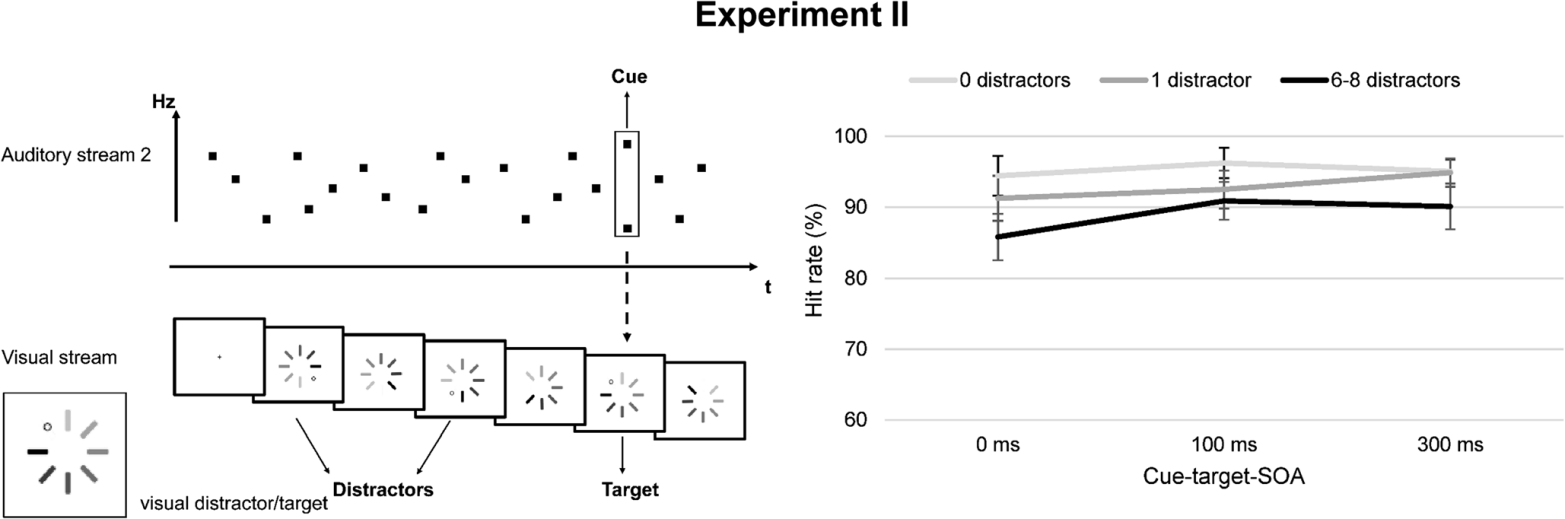
Task design and results of Experiment 2. *Note.* The left panel shows the task design of Experiment 2: Here, the cue was a deviant tone (i.e., overlay of two pure tones) within the stream of pure tones. Target and distractors were identical to Experiment 1. The right panel shows the results of the experiment: Mean hit rates (in %; y-axis) depicted for the stimulus-onset asynchrony (SOA) conditions (x-axis) and the number of distractors. Error bars depict standard errors of the mean. As in Experiment 1, target detection rates were reduced after multiple distractor events at short cue-target SOAs

Stimuli, experimental procedure, and task were identical to Experiment 1 except for the feature defining the auditory cue. In this experiment, the cue was now embedded in stream 2 consisting of sine-wave tones. A deviant tone could appear at one point in this sequence, which consisted of an overlay of two pure tones (1,600 and 2,400 Hz; duration: 30 ms). This deviant tone served as cue. The same auditory cue has been applied in a previous study on the unimodal DID effect (Kern & Niedeggen, 2021a). Importantly, the amplitude of the cue – and therefore its perceived loudness – was matched to the amplitude of all other tones in the RSAP sequence. Thus, the cue was solely defined by the frequency composition. The cue did appear again at a randomized temporal position between 3,100 and 4,000 ms after trial onset. The visual stream containing target and distractors was identical to Experiment 1. The continuous tone was also presented but did not contain rises in amplitude and therefore no task-relevant event. The experimental design is illustrated in Fig. 3.

As in Experiment 1, 290 trials were presented and cue-target SOA (0 vs. 100 vs. 300 ms) and number of distractors (0 vs. 1 vs. 6-8) were manipulated within subjects. The assignment of trial numbers to experimental conditions remained unchanged (see Experiment 1). The first analysis step mirrored the analysis procedure applied in Experiment 1. Additionally, to assess a potential influence of the auditory cue feature, results of Experiments 1 and 2 were analyzed in a two-way ANOVA including the between-subject factor “cue feature” and the within-subject factor “number of distractors,” focusing on SOA 0 ms, where the effect should be most pronounced.

### Results

The results of Experiment 2 are presented in Fig. 3. False alarm rates were low (multiple distractors: *M* = 2.67 %, *SD* = 6.35; without distractors: *M* = .89 %, *SD* = 2.75), and not impacted by distractor presence, *F*(1, 19) = 2.39, *p* = .139, η_p_^2^ = .112.

The auditory cue was detected reliably (*M* = 89.88 %, *SD* = 16.48 for multiple distractors, SOA 0 ms), and cue detection rates did not differ significantly between Experiments 1 and 2 in this critical condition, *F*(1, 37) = 2.02, *p* = .169, η_p_^2^ = .052. Importantly, in none of the experimental conditions including a cue, could significant differences in cue detection rates be stated, indicating a comparable salience of the two cues (see Tables S1 and S2 in the Online Supplementary Material (OSM)).

In replication of the results of Experiment 1, the number of distractors significantly influenced the detection of the subsequent target, *F*(2, 38) = 8.46, *p* = .001, η_p_^2^ = .308. Solely multiple distractors were associated with reduced hit rates (0 vs. multiple distractors: *F*(1, 19) = 11.26, *p* = .003, η_p_^2^ = .372; 1 vs. multiple distractors: *F*(1, 19) = 8.09, *p* = .010, η_p_^2^ = .299; 0 vs. 1 distractor: *F*(1, 19) = 3.19, *p* = .090, η_p_^2^ = .144).

The factor “SOA” had a significant influence, *F*(1.48, 28.27) = 4.65, *p* = .027, η_p_^2^ = .197, in the absence of an interaction between the SOA and number of distractors, *F*(2.31, 43.80) = .98, *p* = .391, η_p_^2^ = .049. Post hoc tests between SOA conditions showed lower hit rates at SOA 0 compared to those at longer cue-target intervals (SOA 0 vs. 100 ms: *F*(1, 19) = 8.11, *p* = .010, η_p_^2^ = .299; SOA 0 vs. 300 ms: *F*(1, 19) = 4.64, *p* = .044, η_p_^2^ = .196). Target detection rates at SOAs 100 and 300 ms did not differ, *F*(1, 19) = .29, *p* = .867, η_p_^2^ = .002.

### Comparison between Experiments 1 and 2: Does the auditory cue feature influence the distractor effect?

Combining the data from Experiments 1 and 2, which solely differed in the feature defining the auditory cue, no main effect of the between-subject factor “cue feature” could be stated, *F*(1, 36) = .23, *p* = .868, η_p_^2^ = .001. In the same way, no interaction between “cue feature” and the within-subject factor “number of distractors” at SOA 0 ms was observed, *F*(2, 72) = .08, *p* = .924, η_p_^2^ = .002 (main effect number of distractors: *F*(1.51, 54.44) = 20.32, *p* < .001, η_p_^2^ = .361).

### Discussion

The influence of distractors on detection of a visual target indicated by an auditory cue observed in Experiment 1 was replicated in Experiment 2. Additionally, Experiment 2 showed that the cue feature rise in amplitude could not account for the high hit rates in Experiment 1 since an auditory cue of constant intensity was associated with a commensurate DIB effect. Importantly, cue detection was reliable and did not differ significantly between the two experiments (see Tables S1 and S2 in the OSM), speaking for a high and comparable salience of both auditory cues.

The presence of very similar and relatively high target hit rates in both experiments is not compatible with our initial assumption that the rise in amplitude led to an especially effective orientation of attention to the target and release of the inhibition. Instead, the findings indicate that dynamics in the “loudness” of an auditory cue may not systematically influence the efficiency of visual target detection during cross-modal DIB. Based on the model proposed for DIB (Niedeggen et al., 2012), the features shared between target and distractors are crucial for the effect, while the cue feature would not be expected to have a substantial influence. The cue functions as a release signal of the distractor-induced inhibitory process (Kern & Niedeggen, 2021a; Niedeggen et al., 2015) and should therefore only require a sufficient salience. The current data are in line with the assumption of the cue as a release signal.

The smaller absolute size of the cross-modal compared to the unimodal DIB was confirmed in Experiment 2 and could not be attributed to the auditory cue feature. However, as another alternative explanation, one could assume that the target feature local luminance change used in Experiments 1 and 2 might be a reason for the comparably weak distractor-induced effect. Maybe the local change in luminance is not associated with an efficient feature inhibition? To test this alternative explanation, in Experiment 3, the target was now defined by a color change. This particular feature was associated with a very pronounced DIB effect within the visual modality (Winther & Niedeggen, 2017a; Winther & Niedeggen, 2018).

## Experiment 3

Cross-modal distractor-induced blindness can reliably be stated for the feature appearance of a small shape – associated with a local change in luminance – as demonstrated by the corresponding results of Experiments 1 and 2. Experiment 3 aimed to test whether the feature defining distractors and target might explain the observed high hit rates by investigating a potential influence of the visual target feature on cross-modal DIB. So far, the brief appearance of a small white circle with a black outline was used as target (and distractor) event, being embedded in a “preloader” symbol in grayscale. This new feature should now be compared to the established feature color change, which has proven to be very efficient in eliciting feature inhibition (Winther & Niedeggen, 2017a; Winther & Niedeggen, 2018), leading to a larger DIB compared to motion stimuli. These previously observed differences, which might rely on differential processing of visual features in the cortex (i.e., ventral vs. dorsal stream) (Milner & Goodale, 2008), revealed a certain degree of feature specificity of visual DIB (Winther & Niedeggen, 2017a).

A brief color change of the preloader symbol from a default color (here: green) to a target color (here: pink) served as target event in Experiment 3. If a larger cross-modal DIB effect can be observed for the feature color than for a local change in luminance, the weaker cross-modal DIB effect can be attributed to target (and distractor) feature. This would confirm the previously described feature specificity.

### Method

#### Participants

Nineteen participants who had not participated in one of the previous two experiments took part in Experiment 3. Once again, the same inclusion and exclusion criteria and required sample size as in Experiments 1 and 2 applied (see Experiment 1). Following the exclusion of one participant due to unreliable performance during pretest (criterion 1), the final sample consisted of 18 participants (13 women; 18–40 years of age; *M*_*age*_ = 27.65 years, *SD* = 6.74).

#### Stimuli, procedure, and design

**Fig. 4:**
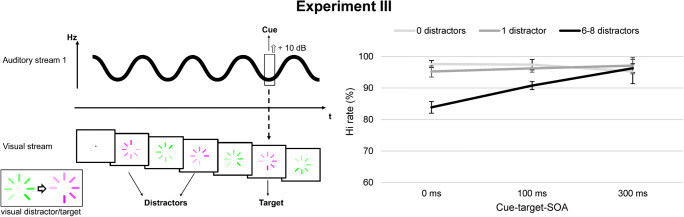
Task design and results of Experiment 3. *Note.* The task design of Experiment 3 is shown in the left panel: The cue was a rise in amplitude as in Experiment 1. In contrast to both previous experiments, the feature defining distractors and target was now a color change of the preloader symbol from green to pink. The right panel illustrates the results: Mean hit rates (in %; y-axis) depicted for the cue-target stimulus-onset asynchronies (SOAs) (x-axis) and the number of distractors. Error bars represent standard errors of the mean. Cross-modal DIB in terms of impaired target detection at short SOAs after multiple distractors could also be stated if distractors and target were defined by a brief color change

The identical stimulus material, procedure, and design as in Experiment 1 was used, with the exception of the configuration of the visual stream. In line with the previous experiments, the visual stream consisted of a “preloader” symbol (1.66° in diameter) with a presentation rate of 10 Hz. The impression of a clockwise motion was once again elicited by varying the luminance of the adjacent bars. While the visual stream contained gray scales ranging from white to black in Experiments 1 and 2, the preloader symbol could now change its color. The preloader was usually colored in green (RGB: [0 255 0]; HSV: [120 100 100]), but could change to pink (RGB: [255 0 255]; HSV: [300 100 100]) for 100 ms. Both colors were adapted from visual DIB studies (Winther & Niedeggen, 2017a; Winther & Niedeggen, 2018). Importantly, color saturation and brightness did not differ between pink and green. The luminance of adjacent bars was graded within the respective color tone to create the impression of clockwise motion. If the color change to pink occurred with or after the auditory cue, this event was the target. Color changes to pink before the cue were considered distractor events that should be ignored. As in Experiment 1, the cue was defined as a short rise in amplitude in the continuous tone. Stream 2 was presented concurrently, but did not contain any task relevant stimuli. The set-up of Experiment 3 is depicted in Fig. 4.

Two hundred and ninety trials were presented. Once again, cue-target SOA (0 vs. 100 vs. 300 ms) and the number of distractors (0 vs. 1 vs. 6–8) were manipulated. Trial numbers for each experimental condition and the statistical analysis performed were identical to Experiments 1 and 2 (see Experiment 1). A potential influence of the between-subject factor “target feature” was examined in a two-way ANOVA with the within-subject factor “number of distractors” and the between-subject factor “target feature” for the cue-target-SOA 0 ms.

### Results

The experimental results of Experiment 3 are displayed in Fig. 4. Response behavior was again reliable and few false alarms were produced (multiple distractors: *M* = 1.93 %, *SD* = 5.32; without distractors: *M* = .56 %, *SD* = 2.36; *F*(1, 17) = 3.13, *p* = .095, η_p_^2^ = .155). Cue detection rates were high (*M* = 97.64 %, *SD* = 3.58 for multiple distractors, SOA 0 ms).

In line with the previous experiments, a significant effect of the number of distractors presented before the cue could be stated, *F*(1.38, 23.48) = 9.09, *p* = .003, η_p_^2^ = .349. Multiple color distractors impaired target detection (0 vs. multiple distractors: *F*(1, 17) = 12.19, *p* = .003, η_p_^2^ = .418; 1 vs. multiple distractors: *F*(1, 17) = 8.57, *p* = .009, η_p_^2^ = .335; 0 vs. 1 distractor: *F*(1, 17) = .33, *p* = .573, η_p_^2^ = .019).

Higher hit rates were observed at larger cue-target-SOAs, *F*(1.51, 25.74) = 10.21, *p* = .001, η_p_^2^ = .375 (SOA 0 vs. 100 ms: *F*(1, 17) = 10.15, *p* = .005, η_p_^2^ = .374; SOA 0 vs. SOA 300 ms: *F*(1, 17) = 12.77, *p* = .002, η_p_^2^ = .429; SOA 100 vs. 300 ms: *F*(1, 17) = 3.99, *p* = .062, η_p_^2^ = .190). Post hoc tests following the significant interaction number of distractors x SOA, *F*(2.50, 42.49) = 7.08, *p* = .001, η_p_^2^ = .294, showed a significant distractor effect at SOAs 0 and 100 ms (SOA 0 ms: *F*(1.32, 22.38) = 11.22, *p* = .001, η_p_^2^ = .398; SOA 100 ms: *F*(2, 34) = 5.11, *p* = .011, η_p_^2^ = .231). At SOA 300 ms. this distractor effect had vanished (*F*(2, 34) = .83, *p* = .446, η_p_^2^ = .046). In the multiple distractor condition, hit rates significantly increased with increasing cue-target-SOA, *F*(1.40, 23.86) = 13.39, *p* < .001, η_p_^2^ = .441, following a linear trend, *F*(1, 17) = 16.16, *p* < .001, η_p_^2^ = .487.

### Comparison between Experiments 1 and 3: Does the visual target feature influence the distractor effect?

Experiments 1 and 3 only differed in the visual feature defining target and distractors and were compared to assess a possible influence of target feature on the cross-modal DIB effect. Importantly, the between-subject factor “target feature” had no significant effect, *F*(1, 34) = .10, *p* = .753, η_p_^2^ = .003. No indications of feature specificity were obtained as demonstrated by the lack of a significant interaction between “target feature” and “number of distractors”, *F*(2, 68) = 1.09, *p* = .342, η_p_^2^ = .031 (main effect number of distractors: *F*(1.46, 49.54) = 22.66, *p* < .001, η_p_^2^ = .400).

### Discussion

The results of Experiment 3 confirmed the cross-modal DIB effect with the typical behavioral characteristics for a different target feature. Importantly, a brief color change elicited a distractor effect of comparable magnitude as a change in luminance accompanying the appearance of a small white circle in Experiments 1 and 2. The findings of Experiment 3 therefore contradict the notion that the overall high hit rates can be attributed to the new target feature but show that they persist for the established feature color change. In contrast to the unimodal DIB, we found no indications for a feature specificity of the cross-modal effect. Consequently, the finding of a relatively small cross-modal distractor effect elicited by a feature that was associated with a large “blindness” in the visual modality (Winther & Niedeggen, 2018) speaks for substantial differences between target detection in purely visual and cross-modal settings. These differences appear to be linked to the modality the cue is presented in: an auditory cue presumably supports visual target detection at short cue-target SOAs more efficiently than a visual cue.

In sum, the observation of a highly significant but comparably small decrease in target detection in the multiple distractor condition in all experiments indicates that despite the distractor-driven inhibitory process, visual target detection appears to succeed more often in cross- than in unimodal conditions.

## General discussion

### Summary of results

In three behavioral experiments, a consistent effect of multiple visual distractors on the detection of a visual target, which was indicated by an auditory cue, was found. Confirming cross-modal distractor-induced blindness, target detection was most affected if cue and target appeared simultaneously and the transient “blindness” had mostly vanished at a cue-target SOA of 300 ms. Furthermore, it was demonstrated that the appearance of a small circle, associated with a local change in luminance, also elicits the inhibitory process underlying DIB, establishing a new visual feature. However, the cross-modal distractor effect was smaller than previously observed within the visual modality, as well as for auditory target detection in unimodal and cross-modal conditions. The expression of the cross-modal DIB did not depend on either the feature of the auditory cue (increase in amplitude vs. frequency composition), or the feature of the visual target (local luminance change vs. color change). Implications of the experimental results are discussed in the following.

### Implications for the inhibitory model underlying distractor-induced blindness

Multiple distractors, sharing the features of a target event, hinder target detection in the visual (Michael et al., 2011; Sahraie et al., 2001) and auditory modality (Kern & Niedeggen, 2021a), as well as in cross-modal conditions with cue and target stemming from different sensory modalities (Kern & Niedeggen, 2021b). The current data provide reliable evidence that, in line with the hypothesis, this distractor effect can also be observed if an auditory cue indicates the appearance of a visual target. Consequently, for all four possible combinations of cue and target within vision and audition, an effect of distractors on target perception can be stated. Independent of the characteristics of cue and target, it was consistently observed that (1) multiple distractors are required to decrease hit rates, while one distractor is not sufficient, and (2) that the largest distractor effect occurs at a cue-target SOA of 0 ms and decreases with increasing temporal distance between cue and target.

Experiments 1 and 2 confirmed a new visual feature, the appearance of a small circle, associated with a local change in luminance, for the DIB effect, therefore extending previous findings on distractor effects for motion, orientation, and color (Michael et al., 2011; Winther & Niedeggen, 2018). That the distractor effect can be elicited by a variety of features, provided they are shared between distractors and target, indicates a large amount of feature adaptability for DIB.

In sum, these findings are in line with the assumption that DIB is caused by the cumulative activation of a negative attentional set by the repeated presentation of task-irrelevant, but target-like stimuli (Hesselmann et al., 2006; Niedeggen et al., 2012). Consequently, the same inhibitory mechanism seems to apply within visual and auditory modality, as well as in both possible combinations of these modalities.However, the current findings also provide extensions of the DIB model. While cue salience can influence the expression of the distractor effect (Hay et al., 2006), Experiments 1 and 2 gave first evidence that the (auditory) cue feature does not seem to impact the efficiency of the distractor-induced inhibition when perceptual load and salience are kept constant. The cue appears to function as a signal for the deactivation of the distractor template, which requires a sufficient salience of the cue but apparently no specific auditory feature. This indicates in combination with previous results that the occurrence of the distractor effect is neither dependent on cue and target occurring in the same modality (Kern & Niedeggen, 2021b) nor on the feature defining the cue. It remains to be examined whether comparable distractor effects for different cue features can also be observed in unimodal settings (e.g., visual), which would be expected based on the DIB model.

Another addition to the model lies in potential differences in the feature specificity of the effect between uni- and cross-modal DIB. For the visual modality, larger inhibitory effects for the target/distractor feature color than for motion stimuli were found, indicating some amount of feature specificity of inhibitory processes (Ariga & Kawahara, 2004; Winther & Niedeggen, 2017a; Winther & Niedeggen, 2018). In contrast, for cross-modal DIB, the current data revealed that a color change is apparently as efficient as a local change in luminance in eliciting a target detection deficit. Whether different visual features are associated with inhibitory effects of different sizes might rely on the modality the cue is presented in. During unimodal DIB, a visual dual task, associated with high perceptual load in the visual modality, is presented. During cross-modal DIB, the dual task consists of one visual and one task-relevant auditory stream, which could be linked to a lower perceptual load and more available resources within each modality (Arrighi et al., 2011; Keitel et al., 2013). Consequently, it could be very cautiously proposed that feature-specific effects regarding the inhibitory process might only be observable under high load conditions, and do therefore not occur in cross-modal tasks. Alternatively, instead of differences between unimodal and cross-modal processing, the observed lack of feature specificity for cross-modal DIB might be attributed to a similar functioning of feature inhibition for all properties that are processed in the same visual pathway. A color change and the appearance of a small circle are both features that are predominantly processed in the ventral visual stream (Kravitz et al., 2013). However, differences in inhibitory processes between predominantly “ventral” and “dorsal” features, as observed for visual DIB (Winther & Niedeggen, 2018), may exist, while taking into account that both pathways are interconnected (Goodale & Milner, 2010; Kravitz et al., 2013; Schenk & McIntosh, 2010). Both preliminary explanations require examination in future studies to pinpoint which account is more fitting.

### Modality-specific differences in the magnitude of the distractor effect

Crucially, the current data allow a comparison of the magnitude of distractor-induced blindness and deafness in uni- and cross-modal conditions. In the visual domain, distractor effects on hit rate (here defined as: zero distractor – multiple distractor condition at cue-target-SOA 0 ms) are very pronounced (i.e., 30 % for orientation stimuli (Niedeggen et al., 2012); 46% for color stimuli (Winther & Niedeggen, 2017a); 27% for motion stimuli (Michael et al., 2011)). In comparison to the visual effect, distractor-induced deafness shows a smaller magnitude. Unimodal and cross-modal DID appear to be roughly comparable in size (unimodal: 8–23% (Kern & Niedeggen, 2021a); cross-modal: 13–28% (Kern & Niedeggen, 2021b)). The current findings in a cross-modal set-up with an auditory cue and a visual target suggest that under these circumstances, the distractor-induced target detection deficit is the smallest (i.e., 9–14%).

The observation of modality-specific differences in the size of the distractor effect raises the question how these differences can be accounted for. First, the high hit rates in the current study might be attributed to a response bias. This explanation can be ruled out, as in all three experiments, the number of false alarms was low (i.e., 0.6–3.5 %), indicating reliable response behavior. Second, it could be assumed that fundamental differences in the processing of distractors between visual and auditory modality exist (e.g., Lavie & Tsal, 1994; Murphy et al., 2013), leading to larger distractor effects in visual compared to auditory modality. However, the data acquired with the DIB/DID paradigm are not in line with this assumption. A third possible explanation concerns the cue: the distractor effect might be more pronounced if the release signal is visual compared to auditory. Strikingly, the previous and current data provide some preliminary evidence for a possible impact of the modality the cue is presented in. The numerically smallest DIB/DID effects were observed if the cue stemmed from the auditory modality. This could indicate that an auditory cue might be an especially effective release signal, possibly due to the function of the auditory modality as an “early warning system” (Dalton & Lavie, 2007). However, the modality of the cue cannot provide a stand-alone explanation since unimodal DID appears to be more pronounced than cross-modal DIB, even though an auditory cue was present both times. Therefore, not only if the modality the cue is presented might it have an impact, but also if the task involves uni- or cross-modal stimulation.

Notably, reduced effects in cross-modal as compared to unimodal tasks have been reported for the related phenomena attentional blink (Arnell & Jenkins, 2004) and inattentional blindness (Sinnett et al., 2006). Arnell and Jenkins (2004) stated that the visual attentional blink appears to be larger than the auditory effect, while cross-modal target detection deficits are usually the smallest (and are often not found at all; see Hein et al., 2006; Soto-Faraco & Spence, 2002; Van der Burg et al., 2007). Strikingly, Arnell and Jenkins (2004) observed that the cross-modal AB at short lags was smaller if a visual second target (T2) followed an auditory first target (T1) than vice versa. In contrast to the typical visual blink and the cross-modal effect for reversed modalities, visual T2 detection after an auditory T1 was enhanced at short compared to long T1- to T2-intervals. This was interpreted as an auditory cueing effect exclusively occurring in this cross-modal condition (Arnell & Jenkins, 2004). This previous finding is in line with our current results and with the assumption that visual target detection succeeds more often if an auditory compared to a visual cue precedes or accompanies the target. During DIB, an auditory cue seems to enable a faster and more efficient allocation of attention to the visual target and a faster release of the negative attentional set than a visual cue stemming from the same sensory modality than the target.

Multisensory research has established that a sound often enhances detection of a visual target, if they occur in temporal proximity (Chang et al., 2015; Fiebelkorn et al., 2011; Frassinetti et al., 2002; Koelewijn et al., 2010; Kusnir et al., 2011; Noesselt et al., 2010; Petersen et al., 2017; Van der Burg et al., 2008). These multisensory enhancement effects have been linked to the integration of temporally overlapping visual and auditory signals into one percept (Koelewijn et al., 2010; Senkowski et al., 2007; Wallace et al., 2020), to a cross-modal spread of attention (Talsma et al., 2010; Tang et al., 2016), and to auditory alerting (Kusnir et al., 2011). Multisensory enhancement effects could provide one suitable explanation for the relatively small cross-modal DIB effect observed here. An attentional enhancement of visual target processing caused by the auditory cue might have counteracted the distractor-driven inhibition of attentional allocation to target features to some degree. Therefore, the distractor effect might have been reduced at short cue-target SOAs compared to unimodal settings.

Taken together, modality-specific differences in the magnitude of the distractor-induced decrease in hit rates can preliminarily be stated. These differences might be attributed to the modality the cue occurs in, with an auditory cue possibly acting as an especially effective release signal of the negative attentional set, and to particularities of cross-modal processing.

### Limitations

One limitation of this study is that the present data do not provide a definite answer to the question as to why cross-modal DIB is a reliable but smaller effect than observed in previous studies. Future research should aim to clarify whether the current findings are due to an auditory enhancement of visual processing, leading to increased hit rates at short cue-target-intervals and an especially efficient release of the negative attentional set, or if alternative explanations are more suitable. One possible alternative explanation might be that a weaker inhibitory process is elicited by distractors in a cross-modal setting. While this explanation appears unlikely since a substantial larger distractor effect was found in a cross-modal task with visual cue and auditory target (Kern & Niedeggen, 2021b), contradicting a general reduction of the distractor-driven inhibition in cross-modal conditions, it cannot be completely ruled out based on the current data. Future research should investigate the strength of the distractor-driven inhibitory process by assessing distractor-evoked event-related potentials (ERPs) in visual, auditory, and cross-modal settings.

Furthermore, it remains unclear why a visual cue did not lead to an enhancement of auditory target detection compared to unimodal DID (Kern & Niedeggen, 2021a, 2021b), speaking for some specificity of multisensory enhancement regarding the modalities cue and target are presented in. It has been reported that a task-irrelevant light can improve auditory perception in contrast to the absence of a visual signal (Lovelace et al., 2003; Odgaard et al., 2004). However, our previous results indicated that a task-relevant visual cue embedded in a RSVP stream does not improve auditory detection in comparison to the unimodal setting (Kern & Niedeggen, 2021a, 2021b). Future studies should examine the modality-specific differences in DIB/DID in more detail to not only improve insight in possible differences in cue and target processing between sensory modalities but also in distinctions between cross-modal settings.

## Conclusion

In sum, it was demonstrated that an inhibitory effect of visual target-like distractors on visual target detection can be stated if the signal for task relevance stems from the auditory modality. The observed cross-modal distractor-induced blindness was reduced in size compared to the cross-modal effect for reversed modalities as well as the unimodal distractor-induced blindness, but exhibited the same characteristics. Since neither cue nor target feature could account for the smaller cross-modal blindness, our results indicate that an auditory cue provides an especially efficient release signal of the inhibited visual features. Compared to a visual signal of task relevance, an auditory cue might lead to a faster attentional allocation to the target stream, especially in cross-modal conditions, where multisensory enhancement effects might boost target detection.

## Supplementary Information

ESM 1 (DOCX 20 kb)
