# Supplementary material for: Are auditory cues special? Evidence from cross-modal distractor-induced blindness
Source: Atten Percept Psychophys. 2022 Jul 28;85(3):889–904. doi: 10.3758/s13414-022-02540-0 (PMC10066119; doi:10.3758/s13414-022-02540-0)
Supplement: Supplementary file 1 — (DOCX 20 kb) [file 13414_2022_2540_MOESM1_ESM.docx]

**Supplementary material to the Manuscript**

**“Are auditory cues special? Evidence from cross-modal distractor-induced blindness” (Kern, L. & Niedeggen, M., 2022)**

**Supplement 1: Cue detection in Experiment I and II**

**Table S1**

*Cue detection rates in Experiment I and II*

|  | Experiment I | | | Experiment II | | |
| --- | --- | --- | --- | --- | --- | --- |
| Distractors | SOA 0 ms | SOA 100 ms | SOA 300 ms | SOA 0 ms | SOA 100 ms | SOA 300 ms |
| 0 | *M* = 95.83 | *M* = 93.61 | *M* = 93.33 | *M* = 94.28 | *M* = 91.67 | *M* = 92.14 |
|  | CI [90.79, 100.87] | CI [87.73, 99.49] | CI [88.32, 98.79] | CI [89.62, 98.95] | CI [86.22, 97.11] | CI [87.50, 96.79] |
| 1 | *M* = 93.89 | *M* = 92.50 | *M* = 93.33 | *M* = 91.91 | *M* = 91.67 | *M* = 93.33 |
|  | CI [88.83, 98.95] | CI [85.78, 99.22] | CI [87.24, 99.42] | CI [87.22, 96.59] | CI [85.44, 97.89] | CI [87.69, 98.97] |
| 6-8 | *M* = 95.69 | *M* = 93.89 | *M* = 92.64 | *M* = 89.88 | *M* = 89.88 | *M* = 90.12 |
|  | CI [89.61, 101.78] | CI [88.98, 98.79] | CI [86.95, 98.37] | CI [84.25, 95.51] | CI [85.34, 94.43] | CI [84.85, 95.39] |

*Note.* Experiment I included a transient rise in amplitude in a continuous tone as cue, while in Experiment II the cue was defined as a deviance in frequency composition in a sequence of sine-wave tones. Mean cue detection rates and 95% confidence intervals (CIs) are provided for each experimental condition containing a cue.

**Table S2**

*Statistical comparison of cue detection rates between Experiment I and II for all experimental conditions including a cue*

|  | Experiment I vs. Experiment II |
| --- | --- |
| SOA 0, 0 distr. | *F*(1, 37)= .21, *p*= .651, η=.006 |
| SOA 0, 1 distr. | *F*(1, 37)= .34, *p*= .563, η=.009 |
| SOA 0, 6-8 distr. | *F*(1, 37)=2.02, *p*= .164, η=.052 |
| SOA 100, 0 distr. | *F*(1, 37)= .24, *p*= .626, η=.006 |
| SOA 100, 1 distr. | *F*(1, 37)= .03, *p*= .855, η=.001 |
| SOA 100, 6-8 distr. | *F*(1, 37)=1.47, *p*= .232, η=.038 |
| SOA 300, 0 distr. | *F*(1, 37)= .13, *p*= .726, η=.003 |
| SOA 300, 1 distr. | *F*(1, 37)= .00, *p*= 1.000, η=.000 |
| SOA 300, 6-8 distr. | *F*(1, 37)= .43, *p*= .514, η=.012 |
| Cue, no target, 0 distr. | *F*(1, 37)= .06, *p*= .800, η=.002 |
| Cue, no target, 6-8 distr. | *F*(1, 37)= .52, *p*= .474, η=.014 |
